# Functional characterisation of the non-essential protein kinases and phosphatases regulating *Aspergillus nidulans* hydrolytic enzyme production

**DOI:** 10.1186/1754-6834-6-91

**Published:** 2013-06-25

**Authors:** Neil Andrew Brown, Paula Fagundes de Gouvea, Nádia Graciele Krohn, Marcela Savoldi, Gustavo Henrique Goldman

**Affiliations:** 1 Faculdade de Ciências Farmacêuticas de Ribeirão Preto, Universidade de São Paulo, São Paulo, Brazil; 2 Laboratório Nacional de Ciência e Tecnologia do Bioetanol (CTBE), Campinas, Brazil

**Keywords:** Carbon catabolite repression, Cellulase, Xylanase, SchA, SnfA

## Abstract

**Background:**

Despite recent advances in the understanding of lignocellulolytic enzyme regulation, less is known about how different carbon sources are sensed and the signaling cascades that result in the adaptation of cellular metabolism and hydrolase secretion. Therefore, the role played by non-essential protein kinases (NPK) and phosphatases (NPP) in the sensing of carbon and/or energetic status was investigated in the model filamentous fungus *Aspergillus nidulans.*

**Results:**

Eleven NPKs and seven NPPs were identified as being involved in cellulase, and in some cases also hemicellulase, production in *A. nidulans*. The regulation of CreA-mediated carbon catabolite repression (CCR) in the parental strain was determined by fluorescence microscopy, utilising a CreA::GFP fusion protein. The sensing of phosphorylated glucose, via the RAS signalling pathway induced CreA repression, while carbon starvation resulted in derepression. Growth on cellulose represented carbon starvation and derepressing conditions. The involvement of the identified NPKs in the regulation of cellulose-induced responses and CreA derepression was assessed by genome-wide transcriptomics (GEO accession 47810). CreA::GFP localisation and the restoration of endocellulase activity via the introduction of the ∆*creA* mutation, was assessed in the NPK-deficient backgrounds. The absence of either the *schA* or *snfA* kinase dramatically reduced cellulose-induced transcriptional responses, including the expression of hydrolytic enzymes and transporters. The mechanism by which these two NPKs controlled gene transcription was identified, as the NPK-deficient mutants were not able to unlock CreA-mediated carbon catabolite repression under derepressing conditions, such as carbon starvation or growth on cellulose.

**Conclusions:**

Collectively, this study identified multiple kinases and phosphatases involved in the sensing of carbon and/or energetic status, while demonstrating the overlapping, synergistic roles of *schA* and *snfA* in the regulation of CreA derepression and hydrolytic enzyme production in *A*. *nidulans.* The importance of a carbon starvation-induced signal for CreA derepression, permitting transcriptional activator binding, appeared paramount for hydrolase secretion.

## Background

Lignocellulolytic fungi secrete a complex arsenal of enzymes that synergistically deconstruct plant cell wall polysaccharides. The capacity of these enzyme cocktails to release utilisable sugars from non-food lignocellulosic material represents an opportunity for the development of a new generation of biofuels, produced directly from plant biomass without the use of extensive pre-treatment. However, efficiencies in industrial enzyme production require dramatic improvement, as the presence of readily metabolisable carbohydrates strongly impedes cellulase and hemicellulase production via carbon catabolite repression (CCR) [1,2]. Genome-wide studies have provided insights into how fungi alter transcription, metabolism and enzyme secretion in response to carbohydrate availability [3-6]. An enhanced understanding of CCR in lignocellulolytic fungi is required for the engineering and exploitation of such regulatory networks to increase enzyme secretion, reducing the costs involved in enzyme production and increasing fermentation efficiency.

In fungi, lignocellulolytic enzyme production is tightly controlled at the transcriptional level by the competitive action of transcriptional activators and repressors [7]. In *Aspergillus nidulans*, *Hypocrea jecorina* and *Neurospora crassa* the orthologous repressors CreA/Cre1 have been shown to block the transcription of genes associated with the utilisation of alternate carbon sources when glucose is present, including cellulolytic and xylanolytic enzymes [8-10]. The *A. nidulans* CreA protein has two Cys_2_His_2_ zinc finger DNA binding structures that demonstrate high similarity to the zinc fingers of the Mig1 repressor involved in *Saccharomyces cerevisiae* CCR [11] and has been demonstrated to be regulated at both the transcriptional and post-translational level [12].

The transcription of genes involved in alternative carbon utilisation also requires the action of transcriptional inducers. In *Aspergilli* the ethanol utilisation pathway is tightly controlled by CreA-mediated CCR and positively induced by the regulon specific transcription factor AlcR. The positive regulator, AlcR, has overlapping binding sites with CreA, suggesting a competitive binding mode of action, while nucleosomal positioning and chromatin organisation has been shown to play a role [13]. Other alternative carbon source genes adopt a similar mechanism of competitive induction including the conserved transcription factors AraR and XlnR, which positively control hemicellulase expression [14]. Interestingly, in *H. jecorina* the cellulase activator, Ace2, has been shown to bind to the same promoter motif as XlnR, while the Hap2/3 complex opens the chromatin structure, promoting nucleosome reassembly and derepression [15]. In *N. crassa* and *A. nidulans*, two newly identified transcription factors, ClrA and ClrB, have been shown to be required for cellulase activity and expression [16].

Sensing the external environment and intracellular energetic status ensures that a fungal organism can balance the requirements for growth and cell survival. *S. cerevisiae* has served as a model organism for the study of such cellular responses. However, many differences are known to exist in filamentous fungi, due to the adoption of different life styles. Protein phosphorylation state represents the most common form of post-translational modification. Protein kinases and phosphatases perform a central role in the transduction of such signals via modulating protein phosphorylation state and activity, thus coordinating subsequent responses. The importance of protein kinases and phosphatases is demonstrated by the fact that 30% of the *S. cerevisiae* genome is modified by these proteins, while collectively kinase and phosphatase genes represent only 6% of the genome [17].

The most well studied examples included the mitogen-activated protein kinases (MAPK), which form the pheromone response, filamentous growth, the osmotic stress response and cell wall integrity pathways. The sensing of glucose or pheromones by the G-protein coupled receptors (GPCRs), results in the activation of the cAMP-protein kinase A (PKA) pathway and the MAPKs cascade, which influence filamentous growth [18]. Intracellularly, glucose is phosphorylated by hexo- and/or gluco-kinases activating *Ras2* signalling that also induces the filamentous growth cAMP-PKA and MAPK pathways [19]. Apart from the well-studied roles in growth, fungal homologues of the *S. cerevisiae* pheromone response/filamentous growth MAPKs have been shown to influence the secretion of hydrolytic enzymes in various plant pathogenic fungi including; *Alternaria brassicicola*, *Cochliobolus heterostrophus* and *Fusarium oxysporum*[20-22].

Homologues of the *S. cerevisiae* sensors of cellular energetic state *Snf1* (sucrose non fermenting) and TOR (target of rapamycin) have been widely identified in filamentous fungi [23-28]*.* The Snf1 has been demonstrated to be required for growth on alternative carbon sources and also regulates the expression of 400 genes in response to carbon exhaustion [29]. The absence of the Snf1 homologue in filamentous fungi, including *Cochliobolus carbonum*, *Ustilago maydis* and *F. oxysporum*, has been shown to reduce hydrolytic enzyme production [24-26]. The essential TOR kinase complexes control cell growth and metabolism in response to environmental cues [30]. TOR has traditionally been related to nitrogen utilisation. However, TOR signalling also influences the expression of AreA-controlled genes involved in carbon metabolism [28]. Therefore TOR may perform dual roles in integrating both carbon and nitrogen signals. Different to the aforementioned kinases, the *A. nidulans* genome contains fewer phosphatases (http://www.aspgd.org), suggesting that each phosphatase demonstrates less specificity and dephosphorylates multiple kinase targets. The known kinase/phosphatase signalling pathways have been proven to be highly conserved and closely interlinked, either regulating one another or the same gene sets, enabling the fine tuning of cellular responses to the extra- and intracellular environment.

Despite recent advances in the understanding of hydrolytic enzyme regulation, less is known about how different carbon sources are sensed and which signalling cascades are subsequently activated, thus coordinating the adaptation of cellular metabolism and hydrolase secretion to the respective carbon source [16,31,32]. Subsequently, this study identified 11 non-essential protein kinases (NPKs) and seven phosphatases (NPPs) involved in cellulase and in some cases also hemicellulase production in *A. nidulans*. The involvement of the identified NPKs in starvation/cellulose-induced responses and CreA derepression was assessed by transcriptomics and fluorescence microscopy of the CreA::GFP in the parental and NPK mutant backgrounds. Collectively, this study demonstrated the overlapping, synergistic roles of the NPKs *schA* and *snfA* in the regulation of CreA derepression and hydrolytic enzyme production. In addition, a period of carbon starvation appeared paramount for CreA derepression and hydrolase induction.

## Results

### Screening the NPKs collection for involvement in cellulase production

**Figure 1:**
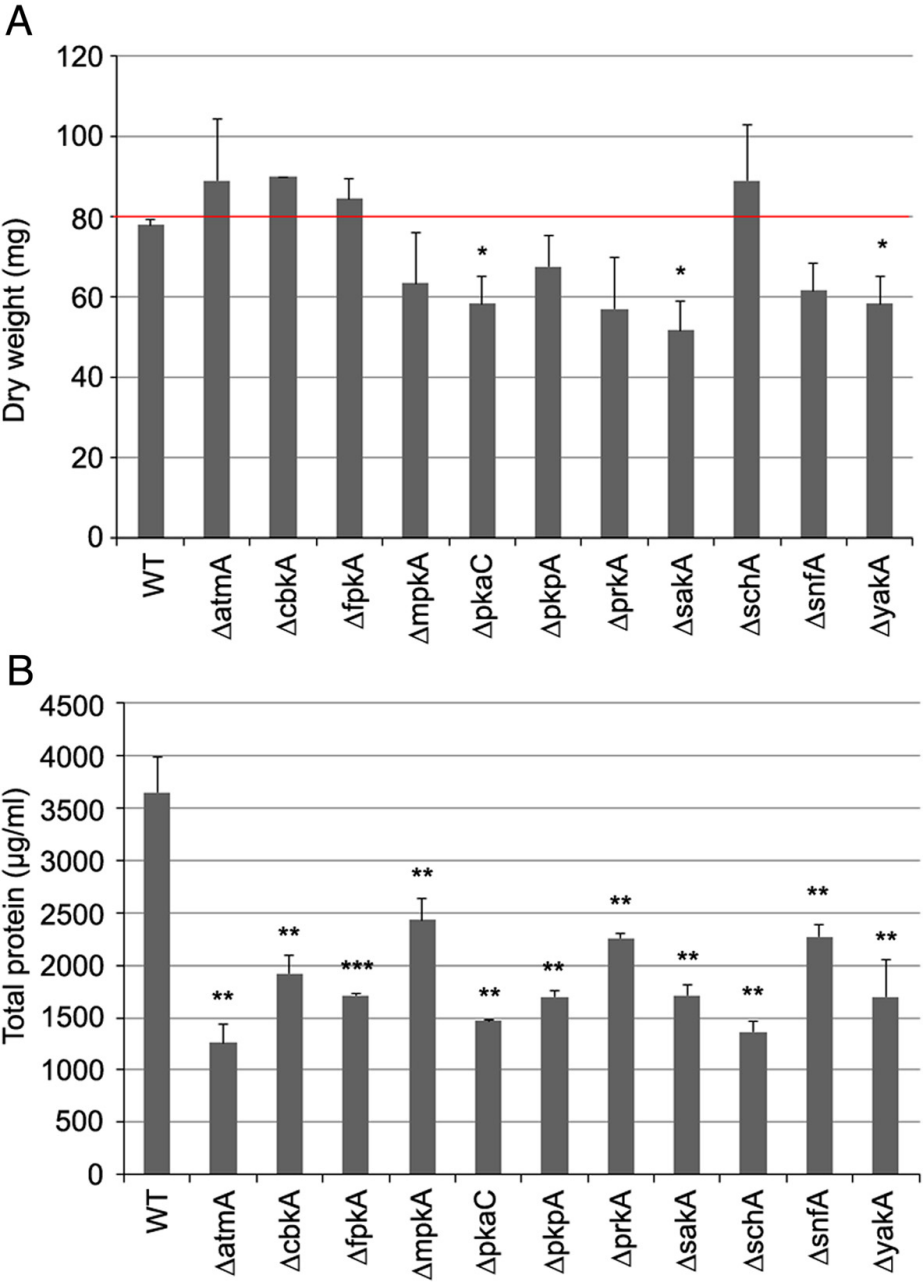
**The AVICEL-specific reduction in growth of eleven NPKs.** The dry weight of the NPKs when grown in complete media **(A)** and the total protein content of the NPKs when grown directly on minimal media plus AVICEL as a sole carbon source **(B)**. One-tailed *t*-test (* *P* <0.05, ** *P* < 0.01, *** *P* < 0.001).

Eleven of the 103 NPKs screened demonstrated a reduction in total protein content when grown in liquid minimal media (MM) supplemented with AVICEL (synthetic cellulose) as the sole carbon source for 10 days, reflecting the reduction in fungal biomass. However, the 11 NPKs did not show a significant reduction in dry weight when cultured in complete media (CM) for 48 h (*p* < 0.01) (Figure 1). The minor reduction in growth of the ∆*pkaC*, ∆*sakA* and ∆*yakA* strains on CM was substantially less than the reduction of growth on MM plus AVICEL when compared to the parental strain. The majority of these 11 NPKs did not demonstrate a reduction in radial growth on solid CM or MM plus AVICEL, except for the ∆*mpkA* and ∆*pkaC* strains which showed extremely decreased radial growth on both solid media (data not shown). The function of these 11 NPKs was determined via homology (BLASTp) with *S. cerevisiae*, if not already characterised in *Aspergilli*, revealing NPKs involved in nutrient/cell energy status sensing and the regulation of cell growth and endocytosis (Table 1). Several of the identified NPKs are known to be involved in the cAMP signalling pathway (*pkaC*, *schA*, *ypkA*), alternative carbon source usage (*snfA*, *sakA*), starvation responses (*atmA*, *pkpA*) and endo- exo-cytosis, polarisation or morphogenesis (*cbkA, fpkA*, *mpkA*, *prkA*).

**Table 1:** The eleven NPKs that demonstrated reduced growth on AVICEL show a conserved theme in nutrient sensing and cell growth

**AspGD**	***A. nidulans *gene name**	**Yeast best hit**	**Function**
AN0038	*AtmA*	*Tel1*	Phospholipid metabolism, DNA damage, cell polarity, starvation
AN10485	Uncharacterised (*CbkA*)	*Cbk1*	RAM signalling, regulates cell morphogenesis
AN0144	Uncharacterised (*FpkA*)	*Fpk1*	Regulates endocytosis, sphingolipid synthesis
AN5666	*MpkA*	*Slt2*	MAP kinase, germination, polarised growth
AN6305	*PkaC*	*Tpk2*	Promotes growth in response to nutrition
AN6207	Uncharacterised (*PkpA*)	*Pkp1*	Negative regulation of pyruvate dehydrogenase
AN10515	Uncharacterised (*PrkA*)	*Prk1*	Actin filament organisation, endocytosis
AN5728	Uncharacterised (*SakA*)	*Sak1*	Upstream activator of SNF1 complex
AN4238	*SchA*	*Sch9*	cAMP signalling, role overlaps with Ras/PKA, regulated by TOR complex
AN7695	Uncharacterised (*SnfA*)	*Snf1*	Required for transcription of glucose repressed genes
AN7104	Uncharacterised (*YakA*)	*Yak1*	Inhibits growth in response to glucose, negatively regulated by Ras/PKA

**Figure 2:**
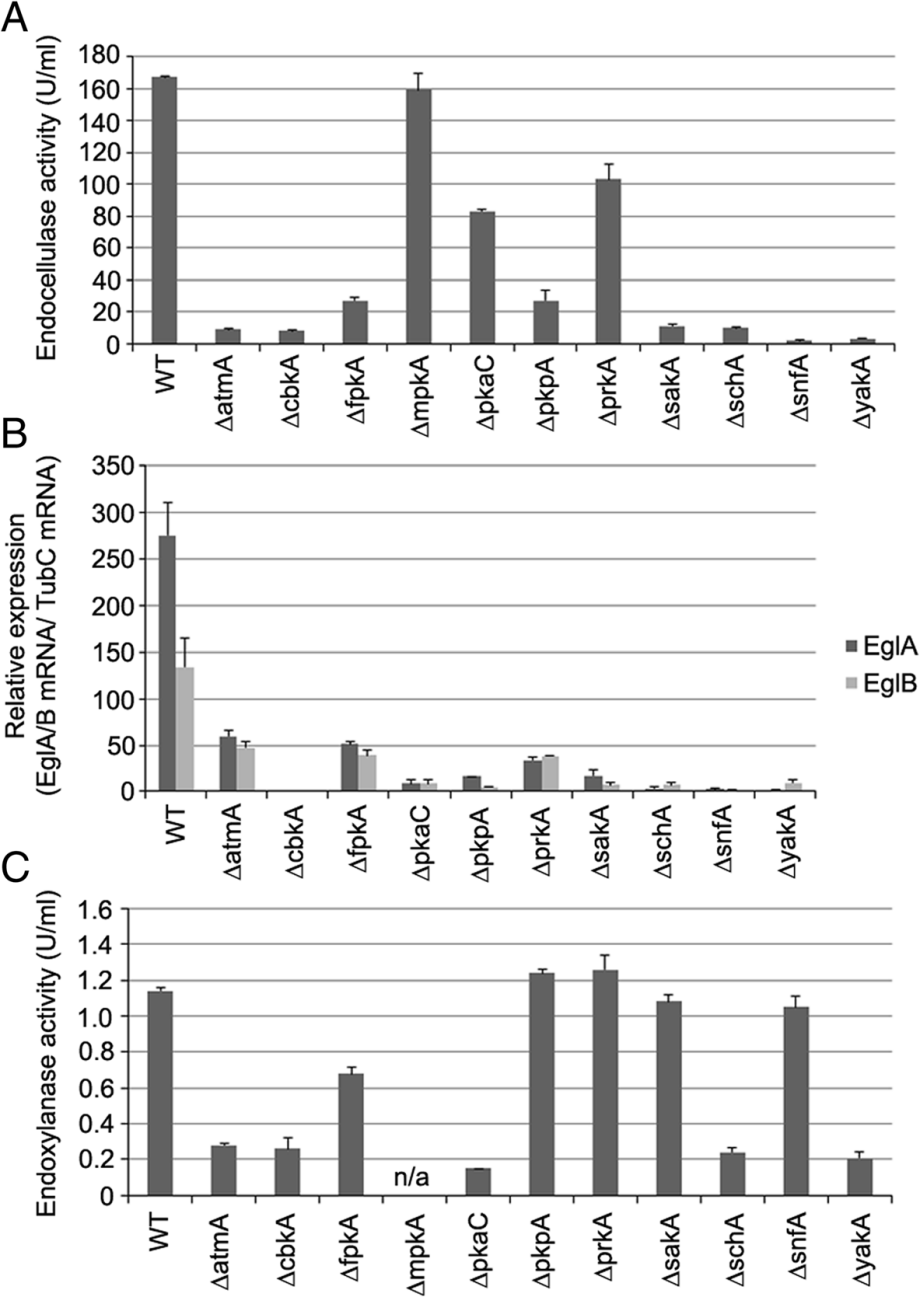
**The identified NPKs mutants demonstrated a reduction in the transcription, and capacity of, hydrolytic enzymes.** The parental (WT), NPKs mutant strains were grown in MM plus 1% fructose overnight and then transferred to AVICEL or xylan as a sole carbon source for an additional 5 or 3 days, respectively. The endocellulase activity of the 5 day AVICEL culture **(A)**. The relative expression of the major endoglucanase genes, *eglA* and *eglB*, in the mycelia isolated from the cultures used to determine endocellulase activity **(B)**. The endoxylanase activity of the xylan cultures **(C)**.

Subsequently, the endocellulase activity of these 11 NPKs and the transcription of two major endoglucanase genes (*eglA/B*) were determined in order to confirm that these NPKs were related to cellulase regulation and/or CCR. Both endocellulase activity and *eglA/B* transcription were dramatically reduced in 10 of the 11 NPKs after 5 days growth in MM plus AVICEL (Figure 2). Only the ∆*mpkA* was shown to have similar to parental levels of endocellulase activity. The ∆*mpkA* strain was subsequently excluded for further analysis. The endoxylanolytic activity of the 10 NPKs grown on xylan for 3 days, separated the NPKs into two groups (Figure 2), those which influenced both cellulase and xylanase activity including the NPKs involved in cAMP signalling (∆*atmA*, ∆*cbkA*, ∆*pkaC*, ∆*schA*, ∆*yakA*) and those which influenced only cellulase production including the NPKs involved in alternative carbon source usage (∆*pkpA*, ∆*prkA*, ∆*sakA*, ∆*snfA*).

### Screening of the non-essential phosphatase (NPP) collection for involvement in cellulase production

**Figure 3:**
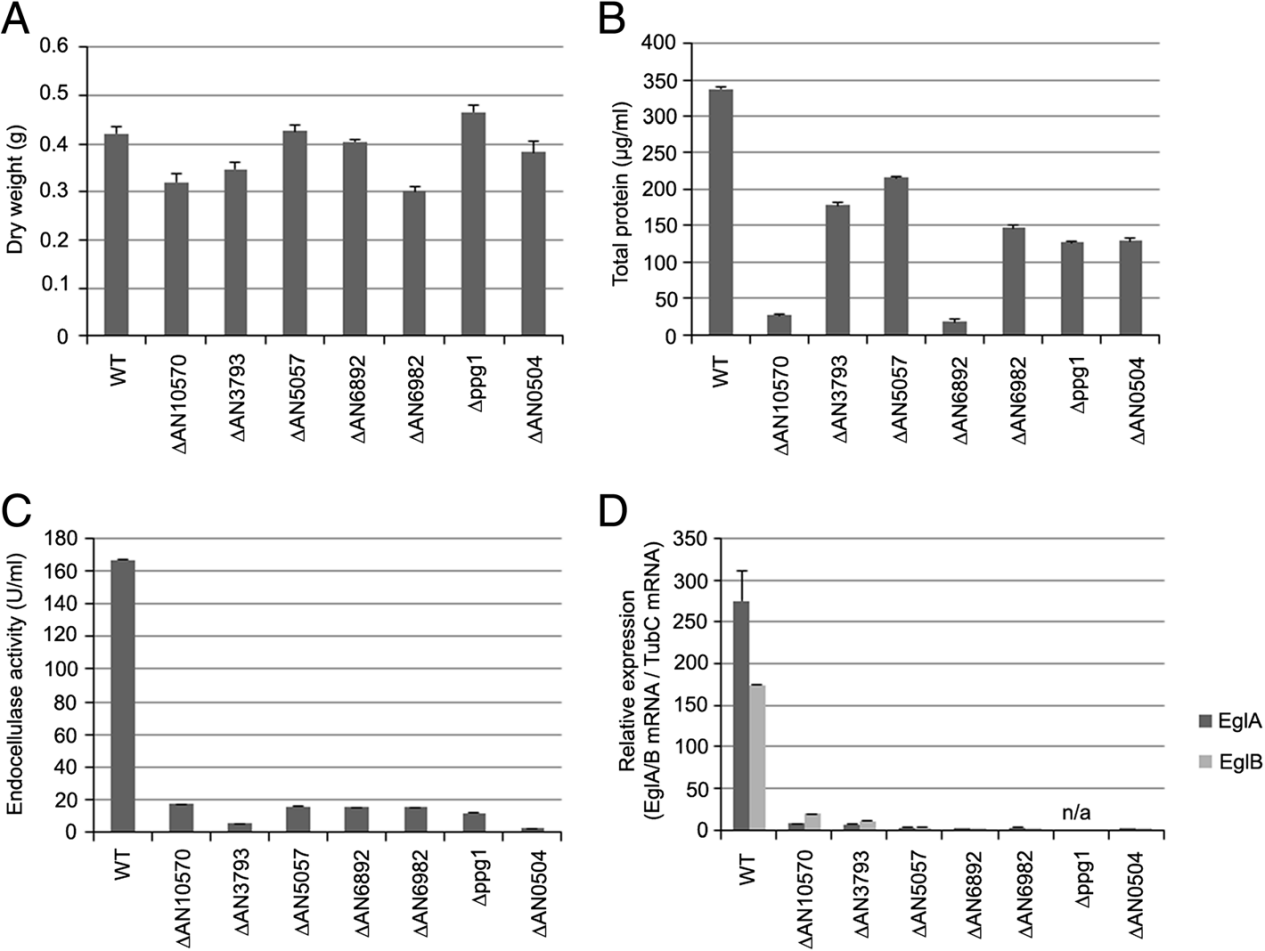
**The seven NPPs identified as being required for growth on AVICEL.** The dry weight of the NPPs when grown in MM plus 2% glucose **(A)** and the total protein content of the NPPs when grown directly on MM plus AVICEL as a sole carbon source **(B)**. The parental (WT) and NPPs mutant strains were grown in MM plus 1% fructose overnight and then transferred to AVICEL as a sole carbon source for an additional 5 days. The endocellulase activity of the AVICEL culture **(C)** and the relative expression of two endoglucanase genes, *eglA* and *eglB*, in the mycelia isolated from the same cultures **(D)**.

The phosphatase collection, containing 28 NPPs, was also screened for reduced growth on AVICEL as a sole carbon source. However, seven NPPs were unable to grow in MM containing glucose or AVICEL (AN0129, AN0914, AN4544, AN4896, AN5722, AN10077 and AN10138), but grew on complete media. Subsequently, it was not possible to study the role of these NPPs in relation to growth on AVICEL as a sole carbon source and hydrolase production. The NPPs that could grow on MM plus glucose were screened for reduced growth on AVICEL. Seven NPPs showed reduced growth on MM plus AVICEL, in terms of total protein, and demonstrated no significant difference in growth on MM plus 2% glucose (Figure 3). The AVICEL specific reduction in growth was correlated with a reduction in endocellulase activity and in the expression of *eglA/B* (Figure 3).

The function of the identified NPPs, if not already characterised in *Aspergilli*, was determined via homology to *S. cerevisiae* (Table 2)*.* Several of the NPPs identified with reduced endocellulase activity had a role in cell cycle (*ltpA*, *cdcA*, *sitA*) and MAPK regulation (*ptcA*, *ptpA*). In *S. cerevisiae* Ppg1A is required for glycogen accumulation, while also associating with Tap42 and Sit4 which are involved in TOR signalling.

**Table 2:** **The functional description of the seven NPPs that demonstrated reduced growth on AVICEL and reduced *eglA/B *transcription**

**AspGD**	***A. nidulans *gene name**	**Yeast best hit**	**Function**
AN10570	Uncharacterised (*LtpA*)	*Ltp1*	Cell cycle
AN3793	*PpzA*	*Ppz1*	Oxidative stress resistance
AN5057	Uncharacterised (*cdcA*)	*Cdc14*	Cell cycle, mitotic exit, meiotic progression
AN6892	Uncharacterised (*PtcA*)	*Ptc1*	Type 2C protein phosphatase, inactivates osmotic stress and activates pheromone response MAPK pathways
AN6982	*PtpA*	*Ptp3*	Inactivates osmotic stress MAPK pathway
AN0164	Uncharacterised (*Ppg1A*)	*Ppg1*	Type 2A-like phosphatase, required for glycogen accumulation, associates with Tap42
AN0504	*SitA*	*Sit4*	G1/S transition of the mitotic cycle, modulates functions mediated by Pkc1p including cell wall and actin cytoskeleton organization

### Protein kinase A (PKA) activity is hyperactivated upon carbon starvation and growth on AVICEL

**Figure 4:**
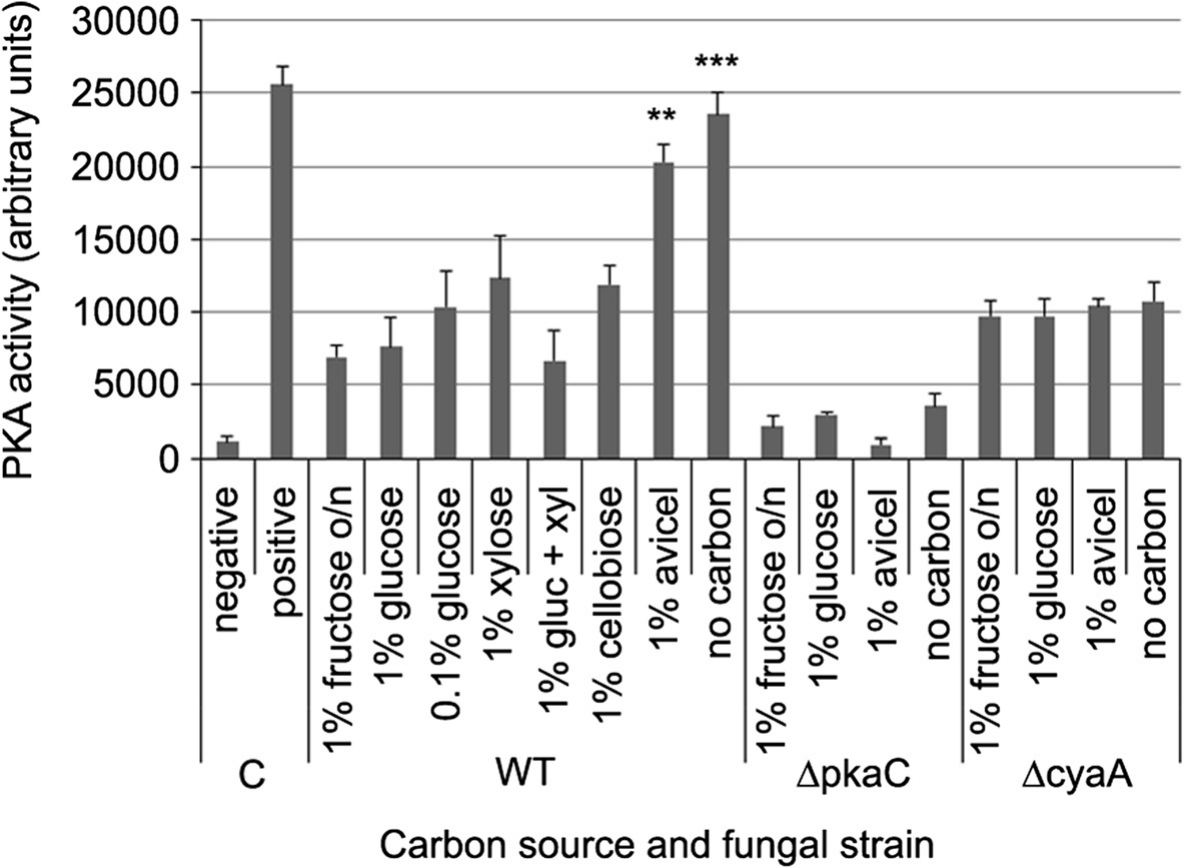
**Protein kinase A was hyperactivated upon growth on AVICEL or carbon starvation.** The parental (WT), ∆*pkaC* and ∆*cyaA* strain were grown in minimal media plus 1% fructose overnight (o/n) and then transferred to various alternative carbon sources for 8 h. The PKA activity from the respective cultures, and the internal cAMP (positive) and water (negative) controls, were determine via densitometry. One-tailed *t*-test (* *P* <0.05, ** *P* < 0.01, *** *P* < 0.001).

The identification of several NPKs involved in the cAMP signalling pathway as being required for both cellulase and xylanase production resulted in an interest to investigate PKA activity in the parental strain in the presence of multiple carbon sources. Overnight 1% fructose MM cultures were transferred to alternative carbon sources for 1–8 h and PKA activity determined. No substantial difference in PKA activity was noted between various carbon sources, except for when the mycelium was transferred to AVICEL or no carbon containing media for 8 h, in which PKA activity approximately doubled (Figure 4). Only a very low, constant level of PKA activity was observed in the ∆*pkaC* mutant on all carbon sources. In the absence of the ability to synthesis cAMP, assessed via utilising the adenylate cyclase ∆*cyaA* mutant [33], an intermediate level of PKA activity was detected in all carbon sources, demonstrating the existence of a cAMP independent route of PKA activation (Figure 4), which may occur under carbon starvation conditions, in part mimicked by the absence of cAMP.

Extracellular glucose is detected via extracellular GCPRs, while intracellular phosphorylated glucose results in RAS activation, with both routes accumulating with adenylate cyclase synthesising cAMP and PKA activation. To confirm that the conventional route of cAMP dependent PKA activation inhibited cellulase production during growth on cellulose, the endocellulase activity of constitutively activated RAS^G17V^ strain [33] was assessed, as this would result in a constant positive signal for repression. The RAS^G17V^ strain demonstrated an approximate eight-fold reduction in endocellulase activity after 5 days growth in MM plus AVICEL (parental, 484 ± 48 U/ml; RAS^G17V^, 60 ± 6 U/ml). This confirmed that the conventional route of PKA activation inhibits cellulase production, while suggesting that starvation induced PKA hyperactivation performed alternative function(s) that directly or indirectly contributed to growth on cellulose.

### Evaluation of CreA nuclear localisation

In order to monitor the dynamics of CCR in *A. nidulans* a CreA::GFP tagged protein under the control of the native promoter was constructed, enabling the study of CreA nuclear localisation under repression/derepression and the evaluation of the signalling components that led to CreA cellular compartmentalisation. The CreA::GFP strain constructed in the present work, demonstrated growth similar to the parental strain on various repressing and derepressing carbon sources (Additional file 1: Figure S1). The microscopic analysis of fluorescence in different carbon sources, including various simple and complex polysaccharides, non-polysaccharides and carbon starvation, assisted in the understanding of the signals involved in CreA nuclear localisation and repression.

**Figure 5:**
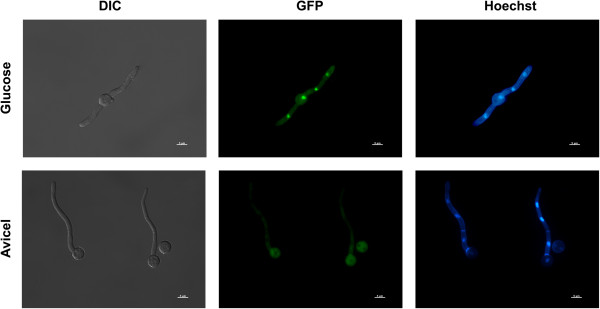
**CreA::GFP localisation alters under repressing and derepressing condition.** Under repressing conditions (glucose 16 h), CreA::GFP constantly localised to the nucleus, while under derepressing conditions (AVICEL 16 h), CreA::GFP was absent from the nucleus. The brightfield (DIC), GFP and the DAPI (for Hoechst nuclear staining) channels are presented. Bar = 5 μm.

The CreA::GFP strain was grown in glucose containing media overnight, which consistently resulted in 100% CreA nuclear localisation (Figure 5), and was then transferred to media containing an alternative carbon source. When the second medium contained a readily metabolisable mono- or di-saccharides, CreA nuclear localisation was high (Table 3). These energy sources are easily taken up and require fewer enzymatic steps prior to entering glycolysis. Within this set, glucose that is phosphorylated and enters directly into glycolysis represented 100% CreA nuclear localisation. Cellobiose, which requires intracellular hydrolysis into glucose, and xylose that enters glycolysis via the pentose phosphate pathway, demonstrated 68% and 70% nuclear localisation respectively. Alternative non-polysaccharide carbon sources, such as glycerol resulted in a far lower level (31%) of nuclear localisation. Complex polysaccharides, such as AVICEL or xylan, represented the lowest level of CreA nuclear localisation. Post 5 h carbon starvation, no CreA nuclear localisation was observed (Figure 5).

**Table 3:** CreA::GFP nuclear localisation post transfer between media containing different carbon sources

**Strain**	**Start culture**	**Nuclear CreA**	**Transfer culture**	**Nuclear CreA**
CreA::GFP	Glucose 16 h (82)	100%	Xylose 2 h (48)	68%
			Cellobiose 2 h (74)	68%
			Xylan 5 h (114)	18%
			AVICEL 5 h (74)	4%
			Glycerol 2 h (32)	31%
			No carbon 5 h (35)	0%
CreA::GFP	Xylose 16 h (24)	70%	Glucose 1 h (20)	100%
CreA::GFP	AVICEL 16 h (22)	0%	Glucose 1 h (30)	100%
CreA::GFP	Glucose 16 h followed by no carbon 5 h (50)	0%	Glucose 1 h (32)	100%
			2-deoxyglucose 1 h (44)	100%
			6-deoxyglucose 1 h (35)	0%
∆*schA*	Glucose 16 h (24)	100%	AVICEL 5 h (31)	83%
∆*snfA*	Glucose 16 h (19)	100%	AVICEL 5 h (28)	76%
∆*atmA*	Glucose 16 h (20)	100%	Avicel 5 h (22)	0%
∆*xprG*	Glucose 16 h (20)	100%	Avicel 5 h (20)	0%

Fungal CreA::GFP nuclear localisation is presented as a percentage of the total number of hyphae evaluated (presented in brackets).

Growing the CreA::GFP strain overnight in glucose (100% nuclear localisation) and then exposing it to carbon starvation for 5 h (~0% nuclear localisation) enabled the study of the positive signals for CreA repression. The addition of 2-deoxyglucose which cannot be successfully metabolised, or 6-deoxyglucose that cannot be phosphorylated, to the carbon starved cultures demonstrated that the positive signal for CreA nuclear localisation required glucose phosphorylation (Table 3).

### Confirmation of NPK involvement in CCR

Sexual crosses between several of the NPK mutants identified from the screening of the kinase collection with either the ∆*creA4* strain [34] or the CreA::GFP strain enabled the confirmation that *schA* and *snfA* were required for CreA derepression and endocellulase production. The absence of *schA* and *snfA* resulted in an inability to remove CreA from the nucleus upon growth on AVICEL (Table 3). Similarly, sexual crosses between ∆*schA* or ∆*snfA* with the ∆*creA4* strain restored the endocellulase activity of these NPKs to parental levels (Additional file 2: Figure S2). However, the absence of *atmA* or the *atmA-*regulated transcription factor *xprG* did not affect CreA nuclear localisation or derepression (Table 3). Collectively, these datasets suggest that *schA* and *snfA* are required for CreA derepression thus permitting cellulase gene induction upon growth on AVICEL, while *atmA* performed additional functions that contributed to cellulase production.

### Microarray analysis revealed the roles performed by *schA* and *snfA* during growth on AVICEL

In response to the transfer to AVICEL containing media for 8 h the parental, ∆*schA* and ∆*snfA* strains modulated the transcription of a similar number of genes, while after 24 h the parental strain showed a far greater transcriptional response, modulating approximately twice as many genes (Additional file 3: Table S1). FetGOat analyses were used to identify the overrepresented GO terms within the differentially expressed genes for each strain. After 8 h culture on AVICEL there was no biological process, cellular component or molecular function overrepresented in the parental strain. After 24 h culture in AVICEL containing media, the parental strain demonstrated an overrepresentation in the modulation of genes involved in aerobic respiration, carbohydrate related catabolic/metabolic processes and ribosomal biogenesis (Table 4, Additional file 4: Table S2). The overrepresentation of multiple ribosomal components was specific to the parental strain. In addition to the processes overrepresented in parental strain, the ∆*schA* strain also showed an overrepresentation of alcohol and quinone metabolic processes, plus the production of energy derived from organic compounds. No biological process, cellular component or molecular function was overrepresented in the ∆*snfA* strain after 24 h growth on AVICEL.

**Table 4:** **The overrepresented GO terms from the FetGOat analysis (*p *<0.05) of the genes differentially expressed within the parental and ∆*schA *strains post transfer from CM to MM plus AVICEL for 24 h**

**Parental**		**∆*schA***	
**Carbohydrate catabolism / metabolism**		**Carbohydrate catabolism / metabolism**	
GO:0016052	carbohydrate catabolism	GO:0044262	carbohydrate metabolism
GO:0030243	cellulose metabolism	GO:0005996	monosaccharide metabolism
GO:0044275	carbohydrate catabolism	GO:0005975	carbohydrate metabolism
GO:0006090	pyruvate metabolism	GO:0046364	monosaccharide biosynthesis
		GO:0016052	carbohydrate catabolism
**Ribosome biogenesis and rRNA processing**		**Respiration and energy production**	
GO:0022613	ribonucleoprotein biogenesis	GO:0046165	alcohol biosynthesis
GO:0000462	maturation of SSU-rRNA	GO:0006066	alcohol metabolism
GO:0000478	rRNA processing	GO:0045333	cellular respiration
GO:0030490	maturation of SSU-rRNA	GO:0009060	aerobic respiration
GO:0042254	ribosome biogenesis	GO:0015980	energy derivation by oxidation of organic compounds
GO:0016072	rRNA metabolism	GO:0006091	generation of precursor metabolites and energy
GO:0006364	rRNA processing		
GO:0000479	rRNA processing	**Quinone biosynthesis**	
GO:0034470	ncRNA processing	GO:0042375	quinone cofactor metabolism
GO:0042274	ribosomal subunit biogenesis	GO:0006744	ubiquinone biosynthesis
GO:0034660	ncRNA metabolism	GO:0006743	ubiquinone metabolism
		GO:0045426	quinone cofactor biosynthesis
**Respiration**		**Cofactors biosynthesis**	
GO:0009060	aerobic respiration	GO:0051188	cofactor biosynthesis
GO:0007005	mitochondrion organization	GO:0051186	cofactor metabolism
**Cofactors biosynthesis**			
GO:0051188	cofactor biosynthesis		
GO:0051186	cofactor metabolism		

Note that no overrepresented GO terms were identified in the ∆*snfA* strain. For the full lists of GO terms refer to Additional file 4: Table S2.

**Figure 6:**
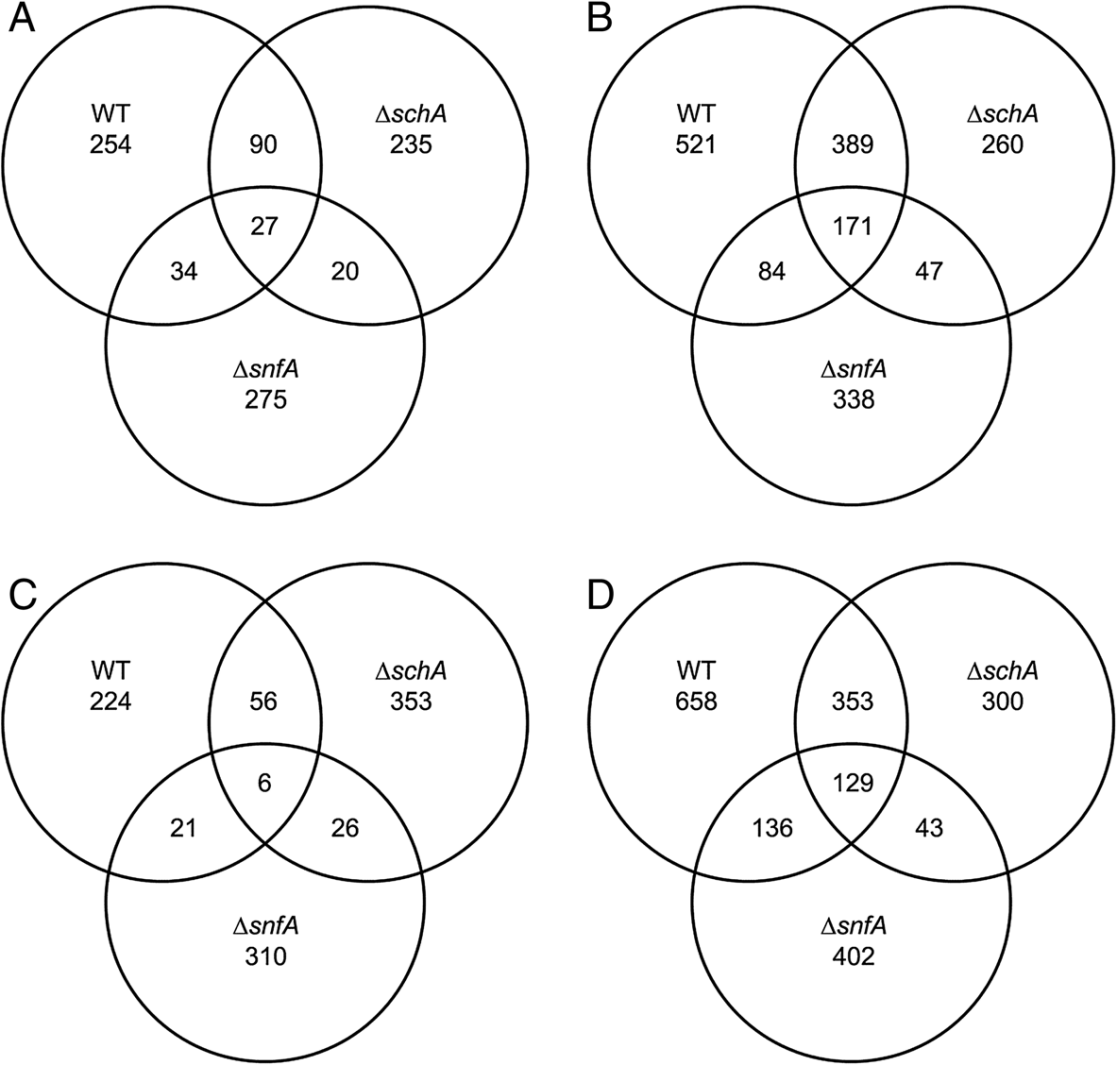
**The identification of the genes modulated by *schA *and *snfA *upon growth on AVICEL.** The venn diagrams for the differentially up **(A,B)** or down **(C,D)** regulated genes (*p* < 0.01) in the parental (WT), ∆*schA* and ∆*snfA* strains post 8 **(A,C)** and 24 h **(B,D)** growth on AVICEL as a sole carbon source.

**Figure 7:**
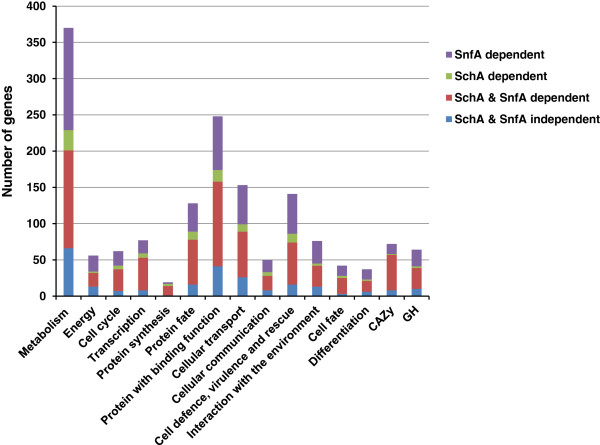
**The contribution of *schA *and *snfA *to AVICEL-induced gene induction.** The distribution of different functional categories and CAZy enzymes, including glycoside hydrolases (GH), among the genes that demonstrated elevated transcription, in the parental strain, post 24 h growth on AVICEL, and whether this induction was dependent on the action of *schA* and/or *snfA*. Note; the near absence of genes independent of *schA* or *snfA* (blue), the substantial contribution of *snfA* represented by the sum of *snfA* dependent (purple) and *snfA* plus *schA* dependent (red) genes, while the majority of *schA* dependent genes overlapped with *snfA* and only a limited number of genes were specifically *schA* dependent responses (green).

A comparison of the differentially up or down regulated genes in the parental and NPK mutant strains again demonstrated that the transcriptome of the parental strain was more distinct from the NPK mutant strain after 24 h culture in the presence of AVICEL (Figure 6), while of the two NPKs mutant strain, ∆*snfA* demonstrated the least similarity to the parental strain. The functional profile of the genes that were induced by 24 h growth in the presence of AVICEL was determined using MIPS functional categories and the CAZy enzyme database (Additional file 5: Table S3). This demonstrated that gene induction in response to growth on AVICEL was greatly dependent on SnfA and to a lesser extent SchA, while SchA showed an overlapping function with SnfA (Figure 7).

**Figure 8:**
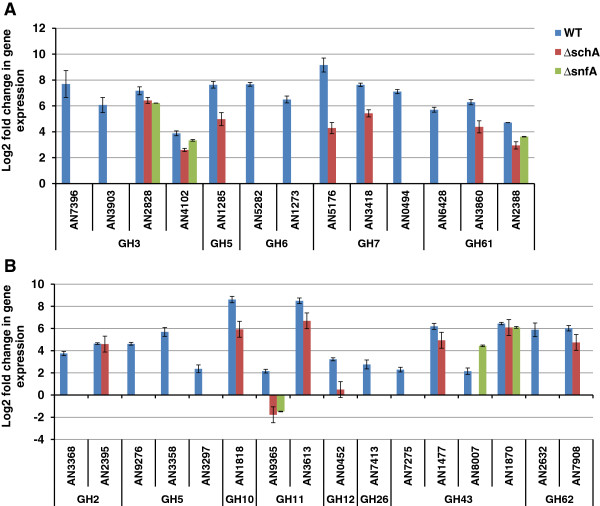
**Cellulase and hemicellulase transcription is reduced in absence of schA or snfA.** The log2 fold change in the expression of a selection of cellulases **(A)** and hemicellulases **(B)** in the parental (WT), ∆*schA* and ∆*snfA* strains post transfer from complete media to minimal media plus AVICEL as a sole carbon source for 24 h. Gene IDs and glycoside hydrolase families are presented.

The CAZy enzymes within these datasets were identified to create a profile of the hydrolytic capacity of each strain. In total, 54 glucoside hydrolases were induced by AVICEL in the parental strain, of which 31 were specifically induced in the parental strain, 8 were induced in all three strains, 14 induced in both the parental and ∆*schA* strains and only one was induced by both the parental and ∆*snfA* strains (Figure 8)*.* Multiple hemicellulase enzymes were also induced by 24 h growth on AVICEL, despite the absence of a specific inducer in the medium. Subsequently, the expression of transcription factors known to be involved with hydrolytic enzyme production was assessed (Additional file 5: Table S3). The expression of *creA* and *araR* did not show differential regulation at either timepoint, in all the three strains. However, *xlnR* was expressed to a higher level in the parental and ∆*snfA* strains after 24 h growth in the presence of AVICEL, but not in the ∆*schA* strain. The induction of *clrB* was substantially greater than that of *clrA* in the parental strain but this induction was absent in the ∆*schA* and ∆*snfA* strains. Additional transcription factors modulated only in the parental strain included a Gcn4p homolog (AN3675), which is involved in glycogen homeostasis, autophagy and starvation stress responses, positive regulators of the ethanol regulon (*alcR*) and acetate utilisation (*facB*). In both of the parental and ∆*schA* transcriptional responses an amino acid starvation (AN1812) and the fatty acid utilisation *farA* transcription factors were also induced.

The modulation of genes involved in transport was assessed. Different groups of transporters were either up regulated upon 24 h growth on AVICEL in all strains (14 transporters), in both the parental and the ∆*schA* strains (33 transporters), in both the parental and ∆*snfA* strains (6 transporters), or specifically in the parental strain (41 transporters). Note the low number of transporters induced in the ∆*snfA* strain. The far majority of the transporters induced in the parental strain lacked a defined function in *A. nidulans.* However, the functionally defined genes included transporters of amino acid, sugar, iron, calcium and sodium (Additional file 5: Table S3). The putative sugar (AN6669) and the alpha glucoside (AN3204) transporters were only up regulated in the parental strain, while an additional putative sugar transporter (AN8737) and a high affinity hexose transporter (AN6923) were induced in both the parental and ∆*schA* strains.

In all strains there was an induction of the *xprG* starvation response transcription factor and *hacA*, which regulates the unfolded protein response, after 24 h growth on AVICEL, suggesting the existence of starvation induced stress. Accordingly, several starvation-related genes, *atg8*, *hxkC* and the GPCR *gprH* were also up regulated. However, despite the induction of *xprG* in all strains these three starvation-response genes were not induced in the ∆*snfA* strain, while the *xprG-*activator kinase gene *atmA* (Krohn N, unpublished results), was repressed at a low level. Other kinase sensors of energetic status including the *sakA* and the *gal83* homolog, which are both required for Snf1 activation and nuclear localisation in *S. cerevisiae*, were only induced in the parental strain, while the TOR kinase (AN5982) was only not induced in the ∆*schA* strain.

Collectively, the transcriptomic data depicts how *schA* and *snfA* are required to regulate the response to carbon limitation and growth on AVICEL. These two NPKs demonstrated a partially overlapping function in the modulation of CAZy enzyme, sugar and amino acid transporters, transcription factors and metabolism, with *snfA* appearing to be of paramount importance.

## Discussion

**Figure 9:**
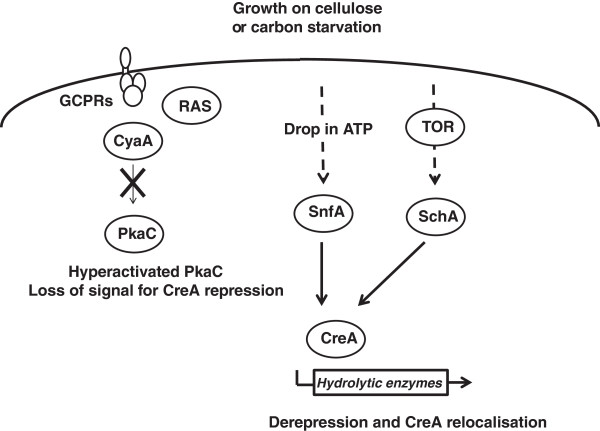
**A schematic for the mechanisms by which NPKs regulate CreA repression and hydrolytic enzyme production.** Upon growth on cellulose as a sole carbon source, a drop in phosphorylated glucose and RAS-mediated PKA activation, results in the loss of the positive signal for CreA nuclear localisation and repression. Subsequently, the drop in intracellular ATP and an alteration in TOR signalling, results in SchA and SnfA controlling CreA re-localisation and hydrolytic enzyme production. Bold arrows represent experimentally proven, while dotted arrow represent hypothetical, connections.

A deep understanding of the mechanisms by which filamentous fungi sense nutrition and cellular energetic status, thus in turn regulating hydrolytic enzyme secretion is paramount for the development of efficient industrial lignocellulosic ethanol production. Protein kinases and phosphatases act as intracellular sensors and perform central roles in numerous signalling cascades that coordinate hyphal growth and metabolism in response to nutrient availability. The presented study identified the protein kinases and phosphatases required for growth on cellulose as a sole carbon source, revealing how different subsets were required for cellulase or both cellulase and hemicellulase production. A modulation of CreA derepression was subsequently identified as the mechanism by two central NPKs, *schA* and *snfA*, controlled hydrolytic enzyme production (Figure 9).

The transcription of lignocellulolytic enzymes is tightly controlled by the competitive action of the CreA repressor and polysaccharide specific inducers. This study demonstrated that carbon starvation resulted in the loss of CreA from the nucleus and derepression, similar to the Mig1 mechanism observed in *S. cerevisiae*[35]. A previous investigation of CreA cellular localisation utilised a fusion protein under the control of a constitutive promoter and implied that intracellular localisation was not involved in the regulation of CreA-mediated repression [36]. Such an approach could overwhelm the system and may have contributed to the differences observed between the two experimental designs. Again similar to *S. cerevisiae*, the absence of the *A. nidulans* Snf1 homolog, which in *S. cerevisiae* is involved in Mig1 nuclear export and alternative carbon usage [35], resulted in a reduced ability of *A. nidulans* to utilise cellulose, transcribe hydrolytic enzymes and relocalise CreA. This appears to be in contrast to *H. jecorina* where the Snf1 homologue has been shown to phosphorylate Mig1 when expressed in *S. cerevisiae* but not the *H. jecorina* counterpart [23], while Cre1 phosphorylation in *H. jecorina* has been shown to positively regulate DNA binding [37]. The similarity between CreA depression during growth on cellulose and carbon starvation, suggested that *A. nidulans* was experiencing starvation under both conditions. The induction of cellulases and hemicellulases when grown on cellulose as a sole carbon source, which would therefore lack a pentose metabolites and a hemicellulase inducer, has also previously been reported under carbon starvation [16] and could be the consequence of CreA derepression rather than polysaccharide specific induction. This is supported by the observation that CreA nuclear localisation was high in the presence of cellobiose, a known inducer of cellulases [38]. This concept suggests that a transient period of starvation is required from CreA derepression, thus liberating the inducer binding sites for gene induction. This is fitting with the model proposed by Delma and colleagues which observed the induction of a subset of hydrolytic enzymes under starvation conditions that were suggested to perform a scouting role [39].

In *S. cerevisiae*, the external sensing of glucose via the GPCRs-PKA pathway and the intracellular phosphorylation of glucose, which results in RAS activation, promotes Mig1-mediated CCR [19]. However, in *A. nidulans* the presence of extracellular glucose proved not to be essential, while glucose phosphorylation was essential, for CreA repression. As shown by the inability to recover CreA nuclear localisation after the addition of the non phosphorylatable 6-deoxyglucose to carbon starved CreA derepressed cultures, while CreA repression was recovered by the addition of the non metabolisable 2-deoxyglucose. The reduced endocellulolytic capacity of the activated RAS^G17V^ strain when grown on cellulose supported the concept that glucose phosphorylation and RAS signalling induces CreA repression. Interestingly, *pkaC* was identified as being required for growth similar to that of the parental strain on cellulose, while PKA activity was hyperactivated during growth on cellulose and carbon starvation. This suggests that the PKA performs additional starvation-induced roles, apart from CreA repression, which in *A. nidulans* that may directly, or indirectly, influence growth and survival on cellulose. During starvation, PKA activity increases in mammalian cells, promoting mitochondrial elongation, preventing autophagic degradation and maintaining ATP production, while the blocking of mitochondrial elongation precipitates in starvation-induced death [40]. In *H. jecorina*, a complex mechanism of light-dependent cellulase regulation has been shown to involve the adenylate cyclase (Acy1) and Pkac1. Schuster *et al.*[41] demonstrated that Acy1 had a positive effect on cellulase gene expression in light and darkness, while Pkac1 only positively influenced cellulase expression in light, but negatively influenced in darkness. Thus in *A. nidulans* PKA may perform additional functions during starvation and growth on cellulose, apart from the typical GPCR/Ras-PKA pathways (Figure 9).

Several additional NPKs involved in the starvation response of multiple organisms, such as the *atmA* and *pkpA* were also identified as being required for growth on cellulose and endocellulolytic enzyme production. In mammalian cells, the pyruvate dehydrogenase complex (PDC), generates NADPH and acetyl CoA from the oxidative decarboxylation of pyruvate and facilitates uptake into the mitochondria. The phosphorylation state of the PDC controls the flux through this irreversible reaction, thus directing metabolism towards the consumption of glucose in respiration or the preservation of glucose for gluconeogenesis [42]*.* Under starvation the pyruvate dehydrogenase kinase phosphorylates and inactivates the PDC conserving glucose and promoting fatty acid utilisation [42]. Therefore, the identification of the *pkpA* kinase suggests that a period of glucose deprivation is experienced by *A. nidulans* when grown on cellulose, which is supported by the observed up regulation of alternative carbon source usage, such as amino acid, ethanol, acetate, fatty acid and cellulose.

The ATM kinase in mammalian cells has been identified as being involved in starvation, insulin signalling and the regulation of glucose homeostasis through the action of the p53 phosphorylation target, which in turn regulates metabolism, mitochondrial respiration and glucose transporters. In addition, a p53-independent ATM pathway has been demonstrated to activate AMPK (the mammalian SnfA homolog) via LKB1 (the mammalian homolog of SakA) via dependent and independent routes [43-45]. Low glycolytic rates and energy stress experienced by *A. nidulans* grown on cellulose could result in a similar AtmA, SakA and SnfA cascade of activation, thus regulating mitochondrial function, sugar uptake, fatty acid utilisation and hydrolytic enzyme production.

The presented study demonstrated how SnfA performed a key role in CreA derepression and the modulation of transcription, metabolism and hydrolytic enzyme secretion. In mammalian and *S. cerevisiae* cells the SnfA homologs have been shown to interact with the essential TOR kinase, which is an integrator of nutrient and growth factor inputs that control cell growth [30]. Interestingly, homologs of two phosphatases revealed in this study to be required for hydrolytic enzyme production and growth on cellulose, are involved in TOR signalling in *S. cerevisiae*. Homologs of the SchA kinases have been shown to operate downstream of TOR [46]. The overlapping but lesser role of the SchA kinase, when compared to SnfA, in the regulation of the transcriptional response to growth on cellulose, in addition to the similar inability to relocalise CreA upon growth on cellulose, suggests that SchA may also have synergistic functions to SnfA in *A. nidulans*, possibly integrating the TOR signal. Similarly in *S. cerevisiae* and *A. nidulans*, the SchA kinase has also been shown to have synergistic and opposing functions with PKA, in the regulation of transcription [33,47].

Multiple NPKs and NPPs involved in filamentous growth, polarisation, and morphogenesis were also identified as being required for growth on cellulose and hydrolase secretion. Endo- and exo-cytosis are key processes for the sensing of, and interaction with, the environment. Such processes have been shown to regulate glucose transporters in mammalian cell [48]. Therefore, the identification of such NPKs and NPPs could represent the impact of these mutations on endo- exo-cytosis, thus influencing secretion, the distribution of transporters in the cell membrane and the import of inducer molecules.

## Conclusion

An initial period of carbon starvation appears to play a role in the activation of the nutrient/energy sensing kinase pathways and that CreA derepression is an essential prerequisite for hydrolytic enzyme induction. Through homology with more extensively studied systems, a model for how the identified kinases and phosphatases regulate hydrolytic enzyme production can be postulated. However, the genetic interactions between these kinases and phosphatases in *A. nidulans* require further study prior to the reconstruction of relevant signalling cascades. Even so, it is clear that SnfA-mediated CreA derepression and transcriptional responses, which regulate metabolism, transport and hydrolytic enzyme secretion, are paramount for growth on cellulose, while the SchA kinase appeared to have an overlapping mechanism and synergistic function with SnfA.

## Methods

### Strains and culture conditions

The parental *A. nidulans* TNO2a3 (*pyrG89; pyroA4; nkuA::argB*) strain was used as a reference in all experiments. The null mutant collections of 103 non-essential protein kinases (NPKs) and 28 non-essential phosphotases, created by *in vivo* recombination in *S. cerevisiae* followed by *A. nidulans* protoplast transformation (http://www.fgsc.net/Aspergillus/KO_Cassettes.htm; [49,50]) were utilised. The ∆*cyaA* and the activated RAS^G17V^ strains were provided by N. Keller, University of Wisconsin-Madison, USA [34]. The ∆CreA strain was provided by M. Flipphi, Instituto de Agroquímica y Tecnología de Alimentos, Spain [34]. All fungal strains were propagated on complete (2% w/v glucose, 0.5% w/v yeast extract, trace elements) or minimal media (1% w/v glucose, nitrate salts, trace elements, pH 6.5) plus 2% w/v agar.

### Screening for reduced growth on AVICEL

Complete media (CM) cultures (50 ml) were inoculated with 1×10^7^ conidia and incubated on a rotary shaker (180 rpm) set at 37°C for 24 h. Liquid minimal media (MM) cultures containing 1% AVICEL, instead of glucose as a carbon source were prepared as with the CM cultures, but were incubated for 10 days under the same conditions. The mycelia of the CM and MM plus AVICEL cultures were filtered, frozen in liquid nitrogen and freeze dried prior to the determination of dry weight and total protein content, respectively. The fungal biomass (dry weight) within the MM plus AVICEL cultures cannot be measured directly, due to the presence of AVICEL. Therefore, total protein content was used as a relative measurement. The mycelia from the AVICEL cultures was ground in liquid nitrogen and added immediately to the protein extraction buffer (Tris base pH 7.5 25 mM, EGTA pH 7.5 15 mM, MnCl_2_ 15 mM, plus a protease inhibitor cocktail (Roche)), vortexed for 5 min prior to centrifugation for 15 min at 14000 g. Protein content was measured using the Bio-Rad protein assay according to manufacturer’s instructions.

### Media shift experiments and enzyme activity assays

Cultures of MM plus 1% fructose (50 ml) were inoculated with 1×10^7^ conidia and incubated on a rotary shaker (180 rpm) set at 37°C for 24 h. Subsequently, the mycelia of the transfer cultures were washed with sterile water, resuspended in liquid MM plus 1% AVICEL or xylan and incubated under the same conditions for 5 or 3 days depending on the respective carbon source. The cultures were filtered and the mycelia frozen in liquid nitrogen prior to being freeze dried for RNA extraction. The culture supernatants were collected for endo- cellulase or xylanase activity assays (Megazyme) according to manufacturer’s instructions.

### Peptag cAMP dependant protein kinase A (PKA) activity assays

Media shift cultures were prepared as described previously. Post washing, the mycelia was transferred to a range of different carbon sources, as stated in the relevant figure and results section, for 8 h. The mycelia from the transfer cultures was collected by filtration, then frozen and ground in liquid nitrogen. Total protein was extracted as described previously and the Peptag cAMP dependent PKA activity assay (Promega) performed according to manufacturer’s instructions. Quantification of the intensity of the phosphorylated substrate was determined via densitometry analysis using the ImageJ software. Results are presented as the total PKA activity per culture.

### Construction of modified strains

The construction of the CreA::GFP strain was performed according to Colot *et al*. [51]. Standard molecular techniques were performed according to Sambrook and Russel [52]. The 5’ untranscribed region (UTR) plus the *creA* gene (minus the stop codon), the *gfp* gene plus a spacer, the *pyrG* gene and the 3’ UTR were co-transformed into *S. cerevisiae*. Homologous recombination within *S. cerevisiae* created the construct, which was subsequently amplified from pooled *S. cerevisiae* DNA, and 20 μg transformed into TNO2a3 according to Osmani *et al*., [53]. Transformants were selected via their ability to grow on solid MM plus pyridoxine in the absence of uridine and uracil. Homologous integration was confirmed via PCR. Sexual crosses between the CreA::GFP or ∆CreA strains with the ∆*schA* and ∆*snfA* strains were confirmed by PCR, using the relevant external forward and exon reverse primers, while the absence of *atmA* was confirmed via increased camptothecin sensitivity (75 μM). The primers used are listed in Additional file 6: Table S4.

### Microscopy

Strains were inoculated onto a coverslip and incubated for 12 h at 25°C in liquid MM plus various carbon sources (1% carbon w/v). During media shift experiments the coverslips were washed with MM lacking a carbon source prior to the addition of the following media and incubated at 25°C, for the duration stated in the text (dependent on carbon source). In the case of 2-deoxyglucose and 6-deoxyglucose, a final concentration 6 mM of either compound was added to 5 h carbon starved cultures and incubated for an additional hour prior to examination. Mycelia mounted on the coverslips were washed with phosphate buffered saline (PBS; 140 mM NaCl, 2 mM KCl, 10 mM NaHPO_4_, 1.8 mM KH_2_PO_4_, pH 7.4). The mycelia were then stained with 100 ng/ml Hoescht 33258 (Molecular Probes) for 2 min. The mycelia were washed again in PBS and examined using a Zeiss epifluorescence microscope with excitations of 359, 498 nm and emissions 461, 516 nm for Hoescht and GFP respectively. Phase contrast bright-field and fluorescent images were captured with AxioCam camera (Carl Zeiss) and processed using the AxioVision software version 3.1.

### RNA extraction and quantitative PCR

Total RNA was isolated using TRIZOL (Invitrogen), treated with DNase (Promega) and purified using the RNeasy® Mini Kit (Qiagen) according to manufacturer’s instructions. RNA integrity was confirmed using the Bioanalyser Nano kit (Agilent technologies) and the Agilent Bioanalyser 2100. Purified RNA was used for cDNA synthesis using Superscript III (Invitrogen) according to manufactures instructions. Quantitative PCRs were performed as previously described [54]. The Taqman fluorescent probes (Invitrogen) used for the endoglucanase genes *eglA* (AN1285) and *eglB* (AN3418) [55] are listed in Additional file 6: Table S4. Expression of the tubulin gene *tubC* (AN6838) was used as an endogenous control.

### Microarray analysis

The parental, ∆*schA* and ∆*snfA* strains (1×10^7^ conidia) were incubated in 50 ml CM on a rotary shaker (180 rpm) set at 37°C for 24 h. The mycelia were washed with sterile water and transferred to MM plus AVICEL for 8 and 24 h. Post incubation, the mycelia was collected by filtration and frozen in liquid nitrogen. Total RNA was extracted and integrity confirmed as described previously. The synthesis of cDNA from 200 ng of RNA and the array hybridisations were performed according to Souza *et al.*[56] with a minor modification to the quantity of RNA used. The RNA isolated from the CM cultures was used as the reference for the MM plus AVICEL of each strain. The dataset was deposited in the Gene Expression Omnibus (http://www.ncbi.nlm.nih.gov/geo/query/acc.cgi?acc=GSE47810).

Genes were determined as differentially expressed between the CM and MM plus AVICEL via a *t*-test (*p* < 0.01) performed within the Mev software [57]. The overrepresented GO terms within the differentially expressed gene sets from each strain were identified using the FetGOat software (http://www.broadinstitute.org/fetgoat/index.html). The differential regulation of gene expression between the three strains post transfer to MM plus AVICEL was determined via venn analysis and the functional profile of the gene lists determined using FunCats (http://pedant.gsf.de/pedant3htmlview/pedant3view?Method=analysis&Db=p3_p130_Asp_nidul). Secreted proteins and hydrolytic enzymes were identified using TargetP (http://www.cbs.dtu.dk/services/TargetP/) and the CAZy enzyme database (http://www.cazy.org/) respectively.

### Statistical analyses

Three biological replicates were performed for all experiments and the statistical tests for significance determined via a one-tailed *t*-test (* *P* <0.05, ** *P* < 0.01, *** *P* < 0.001), unless stated otherwise, using Prism 3.0 (GraphPad).

## Abbreviations

ATP: Adenosine triphosphate; cAMP: Cyclic adenosine monophosphate; CAZy: Carbohydrate active enzymes; CCR: Carbon catabolite repression; CM: Complete media; GFP: Green fluorescent protein; GO: Gene ontology; GPCR: G-protein coupled receptor; MAPK: Mitogen activated protein kinase; MM: Minimal media; NADPH: Nicotinamide adenine dinucleotide phosphate; NPK: Non-essential protein kinase; NPP: Non-essential protein phosphatase; PKA: Protein kinase A complex; PDC: Pyruvate dehydrogenase complex; UTR: Untranscribed region.

## Competing interests

The authors declare that they have no competing interests.

## Author contributions

NAB contributed to the concept, design, acquisition and analysis of data, in addition to the preparation of the manuscript. PG, NK and MS contributed to the acquisition of data, and GHG contributed to the concept and design of the investigation in addition to the preparation of the manuscript. All authors have read and approved the final manuscript.

## Supplementary Material

Additional file 1: Figure S1 The CreA::GFP strain demonstrated a similar phenotype to the parental strain. The CreA::GFP and parental TNO2a3 strains grown on complete media or minimal media for 4 days. Click here for file

Additional file 2: Figure S2 Endocellulase activity was restored when the NPKs kinase mutants were crossed with the ∆*creA* strain. All strains were grown in MM plus 1% fructose overnight and then transferred to AVICEL as a sole carbon source for an additional 5 days. The comparison of the endocellulase activity (U/ml) of the parental, single ∆NPKs and the ∆*creA* strains with the double ∆NPK ∆*creA* strains is presented. Click here for file

Additional file 3: Table S1 The total number of genes determined as differentially expressed (*t-*test p < 0.01) post transfer from CM to MM plus AVICEL for 8/24 h in the parental, ∆*schA* and ∆*snfA* strains. Click here for file

Additional file 4: Table S2 The FetGOat analysis of overrepresented (Fishers exact test, *p* < 0.05) GO terms within the list of genes differentially expressed in the parental and ∆*schA* strains post transfer from complete media to media containing AVICEL as a sole carbon source for 24 h. Note that no GO terms were overrepresented in the ∆*snfA* strain post media transfer. Click here for file

Additional file 5: Table S3 The genes significantly induced (*t* test, *p* < 0.01) post transfer from complete media to minimal media containing AVICEL as the sole carbon source for 24 h in the parental, ∆*schA* and ∆*snfA* strains. Genes are presented according to whether induction occurred in a single or multiple strains. The data presented includes; MIPS functional categorisation, TargetP prediction of secretion and CAZy identification. Click here for file

Additional file 6: TableS4 A list of all the primers used in the presented investigation. Click here for file
