# Supplementary figures and images for: Functional characterisation of the non-essential protein kinases and phosphatases regulating Aspergillus nidulans hydrolytic enzyme production
Source: Biotechnol Biofuels. 2013 Jun 25;6:91. doi: 10.1186/1754-6834-6-91 (PMC3698209; doi:10.1186/1754-6834-6-91)

## Slide 1
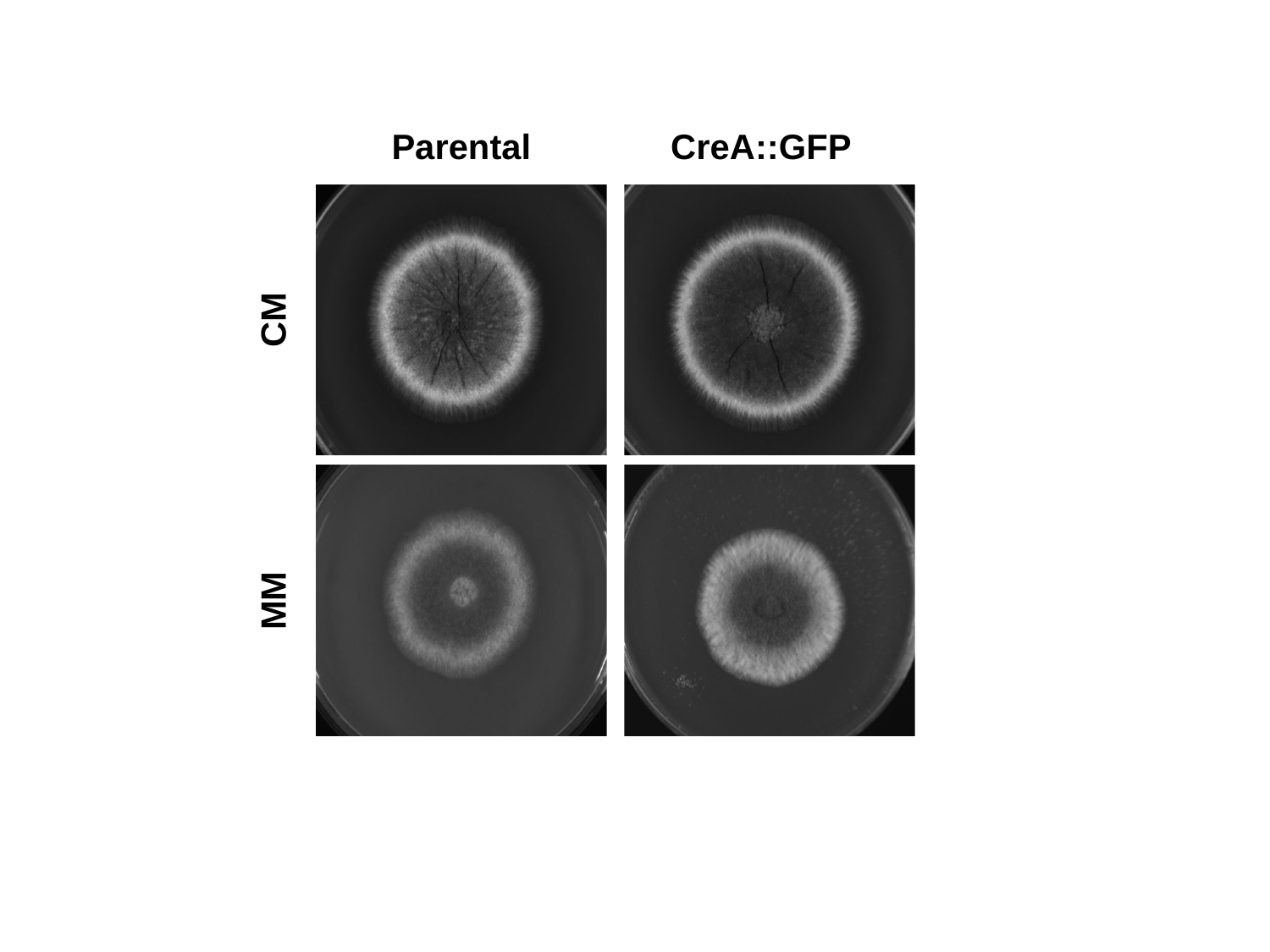

Parental
CreA::GFP
CM
MM

Supplement: Additional file 1: Figure S1 — The CreA::GFP strain demonstrated a similar phenotype to the parental strain. The CreA::GFP and parental TNO2a3 strains grown on complete media or minimal media for 4 days. [file 1754-6834-6-91-S1.pptx]

## Slide 1
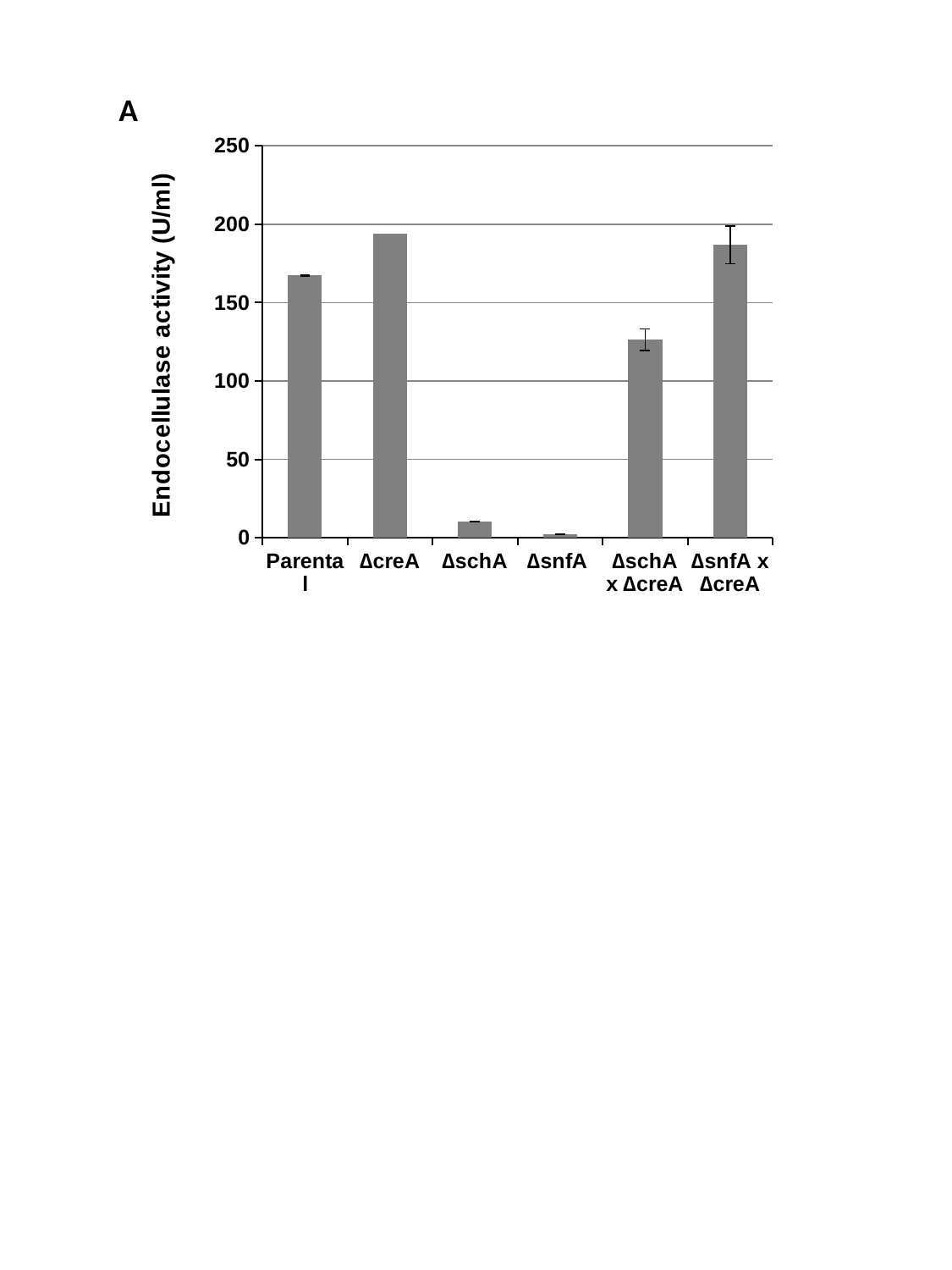

A
### Chart
| Category | Mean |
|---|---|
| Parental | 167.35072463768117 |
| ∆creA | 194.17391304347825 |
| ∆schA | 10.228985507246378 |
| ∆snfA | 2.0478260869565217 |
| ∆schA x ∆creA | 126.27826086956523 |
| ∆snfA x ∆creA | 186.78260869565216 |

Supplement: Additional file 2: Figure S2 — Endocellulase activity was restored when the NPKs kinase mutants were crossed with the ∆creA strain. All strains were grown in MM plus 1% fructose overnight and then transferred to AVICEL as a sole carbon source for an additional 5 days. The comparison of the endocellulase activity (U/ml) of the parental, single ∆NPKs and the ∆creA strains with the double ∆NPK ∆creA strains is presented. [file 1754-6834-6-91-S2.pptx]
