# Supplementary material for: Functional characterisation of the non-essential protein kinases and phosphatases regulating Aspergillus nidulans hydrolytic enzyme production
Source: Biotechnol Biofuels. 2013 Jun 25;6:91. doi: 10.1186/1754-6834-6-91 (PMC3698209; doi:10.1186/1754-6834-6-91)
Supplement: Additional file 3: Table S1 — The total number of genes determined as differentially expressed (t-test p < 0.01) post transfer from CM to MM plus AVICEL for 8/24 h in the parental, ∆schA and ∆snfA strains. [file 1754-6834-6-91-S3.doc]

**Supplementary Table 1** The total number of genes determined as differentially expressed (*t-*test p<0.01) post transfer from CM to MM plus AVICEL for 8/24h in the parental, *∆schA* and *∆snfA* strains.

| Differentially expressed genes | Parental | *∆schA* | *∆snfA* |
| --- | --- | --- | --- |
| All | 712/2387 | 813/1692 | 693/1350 |
| Up regulated | 405/1111 | 372/867 | 356/867 |
| Down regulated | 307/1276 | 441/825 | 337/710 |
