# Supplementary material for: Functional characterisation of the non-essential protein kinases and phosphatases regulating Aspergillus nidulans hydrolytic enzyme production
Source: Biotechnol Biofuels. 2013 Jun 25;6:91. doi: 10.1186/1754-6834-6-91 (PMC3698209; doi:10.1186/1754-6834-6-91)
Supplement: Additional file 6: TableS4 — A list of all the primers used in the presented investigation. [file 1754-6834-6-91-S6.doc]

**Supplementary Table 4 A list of all the primers used in the presented investigation**

| Primer | 5’ – 3’ sequence |
| --- | --- |
| EglA_F | GCCTGCGTCAGTTCTACTATCG |
| EglA_R | CCCCAGTTTCCCGTTCTCT |
| EglB_F | GGAGGCTAATTCGCGGAGTAC |
| EglB_R | CTCAGTTTCCTCCGGCTCATATAAA |
| TubC_F | GCCGTCGCCGAAAGTG |
| TubC_R | TGCACCGATAACGTCGCATTA |
| pRS426-CreA 5UTR_F | GTAACGCCAGGGTTTTCCCAGTCACGACGCTTTTCTTTTTGCCCTTTCG |
| pRS426-CreA 3UTR_R | GCGGTTAACAATTTCTCTCTGGAAACAGCCGTCTGAAAGTACCCCAAGC |
| CreA spacer GFP_R | AGTTCTTCTCCTTTACTCATTCCCCGTGTTCCGAACCTCTCAGCCAAGTCACC |
| PyrG-CreA 3UTR_F | ATTGTTTGAGGCGAATTCTCCGGCCAAAAAACTTCG |
| GFP-PyrG_F | GCATGCAAGCTTGGCGTATTCTGTCTGAGAGGAGGC |
| GFP_R | CTCAGACAGAATACGCCAAGCTTGCATGC |
| PyrG_R | GAATTCGCCTCAAACAATGCTCTTCACC |
| Spacer GFP_F | GGAACACGGGGAATGAGTAAAGGAGAAGAACTTTTCACTGG |
| CreA Insert check | ATAGACATGCCGTCACATGG |
| SchA 5UTR_F | TCTTGAGACGCACATTGACC |
| SchA exon_R | CAGGAGGACGAGATTCTTGC |
